# 异基因造血干细胞移植治疗Shwachman-Diamond综合征：3例报告及文献复习

**DOI:** 10.3760/cma.j.cn121090-20240107-00009

**Published:** 2024-07

**Authors:** 安华 冯, 继敏 施, 华睿 傅, 建 余, 伟燕 郑, 园园 朱, 河 黄, 妍敏 赵

**Affiliations:** 1 浙江大学医学院附属第一医院骨髓移植中心，杭州 310003 Bone Marrow Transplantation Center, the First Affiliated Hospital, Zhejiang University School of Medicine, Hangzhou 310003, China; 2 山东第二医科大学附属医院血液病科，潍坊 261035 Department of Hematology, Affiliated Hospital of Shandong Second Medical University, Weifang 261035, China

## Abstract

研究报告了在浙江大学医学院附属第一医院接受异基因造血干细胞移植（allo-HSCT）的3例Shwachman-Diamond综合征（SDS）患者，并结合相关文献资料总结SDS的临床表现和基因突变特征，探讨allo-HSCT对此类患者的疗效及移植时机。3例SDS患者均为男性，移植年龄分别为32、33、32岁。3例患者均为幼年发病，例1以贫血为首发临床表现，后逐渐进展为全血细胞减少；例2、3均以全血细胞减少为首发临床表现。例1、3有智力障碍，例3有胰腺脂肪化及慢性胰腺炎表现。3例患者均身材矮小。3例患者均检出SBDS:c.258+2T>C剪切位点杂合突变。3例患者的家系成员均无SDS临床表现。3例患者均采用减低剂量预处理方案（氟达拉滨+白消安+司莫司汀+兔抗人胸腺细胞免疫球蛋白）。例1、例2行单倍体造血干细胞移植，例3行无关供者造血干细胞移植。例1移植前诊断骨髓增生异常综合征转化急性髓系白血病，移植后早期复发并死亡；例2为继发性植入不良，血小板输注依赖；例3移植后脱离药物维持治疗，血常规正常。

## Abstract (en)

This study reports on three patients with Shwachman-Diamond syndrome（SDS）who underwent allogeneic hematopoietic stem cell transplantation（allo-HSCT）at the First Affiliated Hospital of Zhejiang University School of Medicine. Based on relevant literature, the clinical manifestations and genetic mutation characteristics of SDS were summarized, and the efficacy and timing of allo HSCT for such patients were explored. Three SDS patients were all male, with transplant ages of 32, 33, and 32 years old, respectively. All three patients were diagnosed in childhood. Case 1 presented with anemia as the initial clinical manifestation, which gradually progressed to a decrease in whole blood cells; Case 2 and 3 both present with a decrease in whole blood cells as the initial clinical manifestation. Case 1 and 3 have intellectual disabilities, while case 3 presents with pancreatic steatosis and chronic pancreatitis. All three patients have short stature. Three patients all detected heterozygous mutations in the SBDS: c.258+2T>C splice site. The family members of the three patients have no clinical manifestations of SDS. All three patients were treated with a reduced dose pre-treatment regimen（Fludarabine+Busulfan+Me-CCNU+ Rabbit Anti-human Thymocyte Globulin）. Case 1 and case 2 underwent haploid hematopoietic stem cell transplantation, while case 3 underwent unrelated donor hematopoietic stem cell transplantation. Case 1 was diagnosed with myelodysplastic syndrome transforming into acute myeloid leukemia before transplantation, but experienced early recurrence and death after transplantation; Case 2 is secondary implantation failure, dependent on platelet transfusion; Case 3 was removed from medication maintenance treatment after transplantation, and blood routine monitoring was normal.

Shwachman-Diamond综合征（SDS）是一种常染色体隐性遗传病，表现为不同程度的骨髓造血异常、胰腺外分泌功能障碍、骨骼畸形以及其他系统异常等。骨髓造血衰竭主要表现为全血细胞减少，可伴有急性髓系白血病（AML）、骨髓增生异常综合征（MDS）等髓系肿瘤转化风险，预后不良。该疾病发生与Shwachman-Bodian-Diamond综合征（SBDS）基因突变密切相关[1]。该基因位于7q11，可以编码一种参与核糖体合成的高度保守的蛋白质。SBDS基因存在40多种突变，c.183-184 TA>CT、c.258 + 2 T>C以及以上两个位点的复合杂合突变是最常见的3种突变形式[2]。另报道有DNAJC21、EFL1和SRP54等基因少见的致病性突变也可以导致SDS[3]–[5]。2018年至2023年期间，3例SBDS基因突变相关血细胞减少患者在浙江大学医学院附属第一医院骨髓移植中心接受异基因造血干细胞移植。

## 病例资料

一、一般情况

研究纳入3例患者，均为男性，出生时间分别为1990年（例1）、1988年（例2）、1987年（例3），移植年龄分别为32、33、32岁，3例患者均为幼年发病，例1以贫血为首发临床表现，后逐渐进展为全血细胞减少；例2、3均以全血细胞减少为首发临床表现。例3有胰腺脂肪化及慢性胰腺炎表现。例1、3有智力障碍。3例患者均身材矮小，婴幼儿期间有脂肪泻病史，治疗期间有反复感染、成分输血史，铁蛋白水平不同程度升高。

二、基因突变

3例均检出SBDS:c.258+2T>C剪切位点杂合突变，例3同时伴有SBDS: c.184A>T:p.K62X。例1同时检出3个血液肿瘤相关基因（TP53、DNAH9、PIM1）错义突变及6个血液肿瘤遗传易感基因（SLX4、SPTB、CTC1、DOCK8、THPO、CFD）错义突变。例2同时检出5个致病证据不充分的基因突变（FANCE、KIT、MTHFD1、PML、ATR）。

三、家族遗传特征

例1父亲检出SBDS: c.258+2T>C剪切位点杂合突变，母亲已故未检测。例2父亲、妹妹及女儿均检出SBDS.c.258+2（IVS2）T>C（NM-016038）杂合突变。例3父亲检出SBDS:c.184A>T无义突变，母亲检出SBDS: c.258+2T>C杂合突变。3例患者家系成员均无SDS临床表现。

四、诊疗经过

例1，男，移植年龄32岁。患者2岁时发现贫血，补铁治疗效果欠佳。后逐渐进展为全血细胞减少，移植前1年（2021年）患者三系水平明显下降，骨髓象：原始细胞占3％，可见病态造血；骨髓流式细胞术：原始髓系细胞1％；二代基因测序示SBDS、TP53基因exon7突变；考虑低增生性MDS。2022年2月复查骨髓涂片示：原幼单细胞占33％；骨髓流式细胞术：原始髓细胞群占25.88％；染色体核型：48,XY,del（4）（q2）,+8,-21,+2mar[1]/50,idem,+12,+mar[1]/46,XY,[2]；考虑MDS转AML。给予CAG（阿糖胞苷+阿柔比星+G-CSF）+维奈克拉+三氧化二砷方案化疗后获得骨髓缓解（BMR）。化疗结束后20天复查骨髓象示原幼单细胞占14％，诊断为病情复发，予挽救性造血干细胞移植。因患者持续粒细胞缺乏合并肛周脓肿，体能状态较差，遂采用减低剂量预处理方案（氟达拉滨30 mg·m^−2^·d^−1^，−10～−5 d；白消安0.8 mg/kg每6 h 1次，−6 d、−5 d；司莫司汀250 mg/m^2^，−4 d；兔抗人胸腺细胞球蛋白总量7.5 mg/kg，−4～−1 d给药）。于2022年4月行父供子单倍体外周血造血干细胞移植，移植后短程甲氨蝶呤、环孢素A（CsA）及霉酚酸酯预防GVHD。单个核细胞（MNC）回输量40.72×10^8^/kg，CD34^+^细胞回输量2.11×10^6^/kg，粒细胞及血小板均于+17 d植入。后患者PLT进行性下降，重组人血小板生成素（TPO）升血小板治疗效果欠佳。+40 d复查骨髓象示BMR，流式细胞术检测微小残留病（MRD）阴性。采用短串联重复序列-聚合酶链反应（STR-PCR）示嵌合度93％（混合嵌合）。移植后2个月血常规：WBC 1.3×10^9^/L，中性粒细胞0.30×10^9^/L，HGB 75 g/L，PLT 5×10^9^/L，骨髓涂片示原幼单细胞占20％，提示移植后早期复发。返回当地医院姑息治疗，最终死亡。

例2，男，移植时33岁。5岁时查体发现全血细胞减少，诊为再生障碍性贫血（AA），不规律服用CsA及中药治疗，病情缓慢进展，间断成分输血支持。2020年1月骨髓穿刺涂片及活检病理提示：增生明显低下（MF-0级）。2022年5月骨髓象：有核细胞量稍减少，粒系增生欠活跃，红系增生明显活跃（病态6％），巨核细胞未见；骨髓活检：增生极度低下，造血组织占<10％。基因检测示SBDS基因杂合突变。于2022年6月行母供子单倍体外周血造血干细胞移植，MNC输注量14.80×10^8^/kg，CD34^+^细胞输注量4.16×10^6^/kg，并输注脐血间充质干细胞1×10^6^/kg。预处理及GVHD预防方案同例1。粒细胞于+16 d植入，血小板于+10 d植入。移植后1个月STR-PCR查嵌合度骨髓血、B细胞、NK细胞均达完全供者嵌合状态（大于95％），T细胞为混合嵌合（82.8％）。移植后2个月复查血常规示三系基本正常；骨髓象：原始细胞比例占3％；STR-PCR示：骨髓血75.8％，T细胞15.8％，B细胞81.9％，NK细胞66.0％。予以供者外周血干细胞输注治疗，STR-PCR示供者型别比例提升（骨髓血96.6％，T细胞57.2％，B细胞88.8％，NK细胞92.3％）。移植后半年余患者感染COVID-19合并堪萨斯分枝杆菌感染，外周血三系进行性下降，呈继发性植入不良，给予抗分枝杆菌治疗、供者外周血干细胞联合脐血间充质干细胞输注、促红细胞生成素以及血小板生成素受体激动剂（TPORA）等治疗，随访至移植后14个月，仍依赖输注血小板。

例3，男，移植时32岁。18岁因反复发热查体示全血细胞减少后因阑尾炎、牙龈炎等多次给予抗感染、升白细胞等治疗。2018年7月血常规示粒细胞缺乏；骨髓象：粒系增生低下，红系明显活跃，幼红细胞及成熟红细胞形态无殊，全片巨核细胞15个。2018年11月复查骨髓象：红系增生明显活跃，内外铁不减少，可见病态造血现象（10％），巨核细胞数量中等，功能差。骨髓活检：造血组织增生低下。全外显子组分析：发现2个SBDS位点突变。2019年3月行无关供者全相合造血干细胞移植。MNC输注量9.50×10^8^/kg，CD34^+^细胞输注量5.25×10^6^/kg，预处理及GVHD预防方案同例1。粒细胞于+11 d植入，血小板于+13 d植入。移植后STR-PCR示完全供者嵌合状态，随访至移植后5年余，脱离药物维持下血象稳定。

## 讨论

SDS是一种罕见的常染色体隐性遗传性疾病，是第四常见的先天性骨髓衰竭性疾病。SDS通常在婴幼儿期起病，具有多器官受累的表现，包括血液系统受累、胰腺外分泌功能不足、骨骼发育和神经发育的异常[6]。骨髓衰竭综合征是导致患者死亡的主要原因。几乎所有SDS患者存在持续性或间断性中性粒细胞减少症，发生反复感染，比如中耳炎、肺炎、败血症等，严重感染可危及生命。贫血和血小板减少也可见，少数患者可出现重型再生障碍性贫血[7]。此外，SDS患者易在骨髓中出现克隆性细胞遗传学异常[8]，高达30％的病例进展为MDS或AML[9]。研究表明，SDS患者由于胰腺功能不全和脂溶性维生素水平降低，导致营养消化吸收不良，进而导致患者生长发育迟缓，同时增加了患者骨质疏松的风险[10]。SDS患者还可出现其他消化系统改变，最突出影响的是肝脏，转氨酶升高和肝脏肿大最为常见，部分患者出现胆汁酸水平升高，胆汁淤积。除此之外，还有部分SDS患者存在神经认知功能的损害，语言能力、感知觉、注意力及学习能力明显受损。本报道中3例患者确诊年龄较晚，均有反复感染、身材矮小等临床表现，例1和例3患者同时伴有智力障碍，例3患者伴有胆汁淤积，慢性胰腺炎等消化系统症状。

SDS相关的突变基因为SBDS，位于染色体7q11。BooCock等[11]证实约90％的患者存在SBDS基因功能缺失性突变，引起核糖体大亚基成熟过程有关的SBDS核糖体蛋白异常，导致SDS的发生。因此，SDS是一个核糖体合成缺陷的疾病。本报道中3例患者均为SBDS:c.258+2T>C剪切位点杂合突变，另外例1检出TP53基因错义点突变。TP53信号通路由核糖体生物合成缺陷和蛋白质翻译异常激活[12]，突变型TP53除了失去其抑癌功能外，还通过获得新的功能活性进一步促进癌症发生。TP53通路激活的改变与胰腺腺泡细胞萎缩、胰腺实质结构改变以及MDS/AML有关。先前的研究表明，TP53突变可见于近50％的SDS患者中，SDS患者会出现频繁的体细胞克隆性造血，携带杂合致病性TP53突变的SDS造血干祖细胞中存在持续核糖体生物合成的应激，诱导第2个TP53等位基因失活的克隆产生并扩增，增加髓系肿瘤转化风险[13]。最近也有报道称，SBDS突变的SDS合并TP53突变的儿童患者预后更差[14]。例1伴TP53突变，呈现低增生MDS向AML迅速转化的特点。

尽管很多SDS患者有血液学表现，但大多数患者不需要移植。性别、首次确诊年龄、血液学参数分布对患者总生存均无明显影响[15]。只有部分SDS患者会进展为严重的全血细胞减少症或转化为恶性肿瘤[16]–[17]。因此，在SDS中不建议采用抢先移植。早期治疗方案以对症支持治疗为主，包括营养调节、抗感染、保肝、祛铁、预防出血等。另外SDS疾病相关的认知及行为障碍会影响患者的生活质量[18]，可依据年龄分层进行神经心理学筛查和干预。

然而，造血干细胞移植仍然是部分发生骨髓衰竭（BMF）或转化为髓系恶性肿瘤的SDS患者的唯一治疗方法[19]，而BMF移植预后明显优于髓系恶性肿瘤转化者。本研究中，例1母亲已故，无关供者及单倍体供者均不可获得，只能选择携带SBDS杂合基因的父亲作为供者。漫长的供者寻找过程使该患者错过治疗移植时机，而在SDS转AML阶段进行了挽救性异基因干细胞移植，移植后复发，最终死亡。

就SDS移植预处理方案强度而言，分为清髓性预处理（MAC）和减低强度预处理。MAC方案包括使用白消安>8 mg/kg（口服或等效静脉剂量）或美法仑>140 mg/m^2^或塞替派>10 mg/kg或分次全身放射治疗≥1 000 cGy。其他方案均为减低强度预处理[19]。对于成人患者，减低强度预处理方案适合重度血细胞减少患者，而对于SDS转化AML/MDS的患者，部分中心进行MAC以降低移植后复发率。然而，无论MAC或者减低强度预处理方案似乎都不能克服TP53突变的不良预后影响[14]。SDS伴TP53突变继发髓系肿瘤仍然是血液学领域需要攻坚的难题。在无合适单倍体供者及无关供者情况下，脐血造血干细胞移植是极好的替代选择[20]–[21]。

血小板减少是造血干细胞移植后常见并发症，发生率为5％～37％。单倍体移植后血小板减少发生率为10.1％[22]，HLA相合供者移植后血小板减少发生率为22.8％[23]。感染、GVHD、复发、植入不良、药物、血栓性微血管病等因素均可引起不同程度的血小板减少[24]–[27]，CD34^+^细胞输注量不足可导致巨核系重建延迟，移植类型与供者特异性抗体也是影响血小板重建的重要因素[28]。近年来发现，新冠病毒感染可能干扰CD34^+^造血干细胞和巨核细胞-红样祖细胞分化，导致血小板减少[29]。本研究例2单倍体移植后出现继发性血小板植入不良，与COVID-19及堪萨斯分枝杆菌感染等因素相关。多中心相关研究证明间充质干细胞输注对治疗移植后血小板减少或植入不良具有一定的疗效[30]–[31]。而rhTPO以及TPO受体激动剂能够促进血小板植入[32]。

综上，随着对SDS临床表型的认识以及基因分析手段的完善，SDS精准诊断日益发展。如进展至严重的全血细胞减少、MDS和AML，预后极差，我们需定期监测患者血常规及骨髓相关检查等，以实现对患者病情的更好监测，或依据不良遗传学信息识别髓系恶性肿瘤高危转化患者，在疾病进展前评估患者是否需要进行移植。而对于已经确诊的髓系恶性肿瘤患者，未来应考虑采用新的治疗方法和移植策略，以改善疾病预后。

## References

[1] Huang JN, Shimamura A (2011). Clinical spectrum and molecular pathophysiology of Shwachman-Diamond syndrome[J]. Curr Opin Hematol.

[2] Nelson AS, Myers KC (2018). Diagnosis, Treatment, and molecular pathology of Shwachman-Diamond syndrome[J]. Hematol Oncol Clin North Am.

[3] Pressato B, Valli R, Marletta C (2012). Deletion of chromosome 20 in bone marrow of patients with Shwachman-Diamond syndrome, loss of the EIF6 gene and benign prognosis[J]. Br J Haematol.

[4] Dhanraj S, Matveev A, Li H (2017). Biallelic mutations in DNAJC21 cause Shwachman-Diamond syndrome[J]. Blood.

[5] Stepensky P, Chacón-Flores M, Kim KH (2017). Mutations in EFL1, an SBDS partner, are associated with infantile pancytopenia, exocrine pancreatic insufficiency and skeletal anomalies in aShwachman-Diamond like syndrome[J]. J Med Genet.

[6] Ginzberg H, Shin J, Ellis L (1999). Shwachman syndrome: phenotypic manifestations of sibling sets and isolated cases in a large patient cohort are similar[J]. J Pediatr.

[7] Cipolli M (2001). Shwachman-Diamond syndrome: clinical phenotypes[J]. Pancreatology.

[8] Dror Y, Durie P, Ginzberg H (2002). Clonal evolution in marrows of patients with Shwachman-Diamond syndrome: a prospective 5-year follow-up study[J]. Exp Hematol.

[9] Donadieu J, Fenneteau O, Beaupain B (2012). Classification of and risk factors for hematologic complications in a French national cohort of 102 patients with Shwachman-Diamond syndrome[J]. Haematologica.

[10] Toiviainen-Salo S, Mäyränpää MK, Durie PR (2007). Shwachman-Diamond syndrome is associated with low-turnover osteoporosis[J]. Bone.

[11] Boocock GRB, Morrison JA, Popovic M (2003). Mutations in SBDS are associated with Shwachman-Diamond syndrome[J]. Nat Genet.

[12] Amundson MJ, Trotter CM (1991). Developing a network of mental health workers for Pacific Islands[J]. Int Nurs Rev.

[13] Kennedy AL, Myers KC, Bowman J (2021). Distinct genetic pathways define pre-malignant versus compensatory clonal hematopoiesis in Shwachman-Diamond syndrome[J]. Nat Commun.

[14] Lindsley RC, Saber W, Mar BG (2017). Prognostic mutations in myelodysplastic syndrome after stem-cell transplantation[J]. N Engl J Med.

[15] 安 文彬, 刘 超, 万 扬 (2020). 伴髓系恶性转化的Shwachman-Diamond综合征患儿的临床特征及基因突变分析[J]. 中国当代儿科杂志.

[16] Donadieu J, Fenneteau O, Beaupain B (2012). Classification of and risk factors for hematologic complications in a French national cohort of 102 patients with Shwachman-Diamond syndrome[J]. Haematologica.

[17] Cada M, Segbefia CI, Klaassen R (2015). The impact of category, cytopathology and cytogenetics on development and progression of clonal and malignant myeloid transformation in inherited bone marrow failure syndromes[J]. Haematologica.

[18] Kerr EN, Ellis L, Dupuis A (2010). The behavioral phenotype of school-age children with shwachman diamond syndrome indicates neurocognitive dysfunction with loss of Shwachman-Bodian-Diamond syndrome gene function[J]. J Pediatr.

[19] Myers K, Hebert K, Antin J (2020). Hematopoietic Stem Cell Transplantation for Shwachman-Diamond Syndrome[J]. Biol Blood Marrow Transplant.

[20] Vibhakar R, Radhi M, Rumelhart S (2005). Successful unrelated umbilical cord blood transplantation in children with Shwachman-Diamond syndrome[J]. Bone Marrow Transplant.

[21] Yoshimura S, Mizuno T, Osumi T (2021). Successful umbilical cord blood transplantation with reduced-intensity conditioning for acute myeloid leukemia in a child with shwachman-diamond syndrome[J]. J Pediatr Hematol Oncol.

[22] Tang FF, Cheng YF, Xu LP (2020). Basiliximab as treatment for steroid-refractory acute graft-versus-host disease in pediatric patients after haploidentical hematopoietic stem cell transplantation[J]. Biol Blood Marrow Transplant.

[23] Akahoshi Y, Kimura SI, Gomyo A (2018). Delayed platelet recovery after allogeneic hematopoietic stem cell transplantation: Association with chronic graft-versus-host disease and survival outcome[J]. Hematol Oncol.

[24] First LR, Smith BR, Lipton J (1985). Isolated thrombocytopenia after allogeneic bone marrow transplantation: existence of transient and chronic thrombocytopenic syndromes[J]. Blood.

[25] Bruno B, Gooley T, Sullivan KM (2001). Secondary failure of platelet recovery after hematopoietic stem cell transplantation[J]. Biol Blood Marrow Transplant.

[26] Dominietto A, Raiola AM, Van lint MT (2001). Factors influencing haematological recovery after allogeneic haemopoietic stem cell transplants: graft-versus-host disease, donor type, cytomegalovirus infections and cell dose[J]. Br J Haematol.

[27] Zhao YM, Gao F, Shi JM (2019). Incidence, risk factors, and outcomes of primary poor graft function after allogeneic hematopoietic stem cell transplantation[J]. Biol Blood Marrow Transplant.

[28] Zhao XS, Zhao XY, Huo MR (2017). Donor-specific anti-human leukocyte antigen antibodies predict prolonged isolated thrombocytopenia and inferior outcomes of haploidentical hematopoietic stem cell transplantation[J]. J Immunol Res.

[29] Balzanelli MG, Distratis P, Dipalma G (2021). Sars-CoV-2 virus infection may interfere CD34+ hematopoietic stem cells and megakaryocyte-erythroid progenitors differentiation contributing to platelet defection towards insurgence of thrombocytopenia and thrombophilia[J]. Microorganisms.

[30] Servais S, Baron F, Lechanteur C (2023). Multipotent mesenchymal stromal cells as treatment for poor graft function after allogeneic hematopoietic cell transplantation: A multicenter prospective analysis[J]. Front Immunol.

[31] Zhu LD, Liu J, Kong PY (2022). Analysis of the efficacy and safety of avatrombopag combined with MSCs for the treatment of thrombocytopenia after allogeneic hematopoietic stem cell transplantation[J]. Front Immunol.

[32] Gao F, Zhou XY, Shi JM (2020). Eltrombopag treatment promotes platelet recovery and reduces platelet transfusion for patients with post-transplantation thrombocytopenia[J]. Ann Hematol.

